# The role and prospects of telemedicine in the treatment of heart failure patients: a narrative review

**DOI:** 10.12771/emj.2025.00360

**Published:** 2025-04-08

**Authors:** Dong-Ju Choi

**Affiliations:** Division of Cardiology, Cardiovascular Center, Department of Internal Medicine, Seoul National University Bundang Hospital, Seoul National University College of Medicine, Seongnam, Korea

**Keywords:** Digital technology, Heart failure, Medication adherence, Patient-centered care, Telemedicine

## Abstract

Heart failure (HF) represents a significant global health burden characterized by high morbidity, mortality, and healthcare utilization. Traditional in-person care models face considerable limitations in providing continuous monitoring and timely interventions for HF patients. Telemedicine—defined as the remote delivery of healthcare via information and communication technologies—has emerged as a promising solution to these challenges. This review examines the evolution, current applications, clinical evidence, limitations, and future directions of telemedicine in HF management. Evidence from randomized controlled trials and meta-analyses indicates that telemedicine interventions can improve guideline-directed medical therapy implementation, reduce hospitalization rates, improve patient engagement, and potentially decrease mortality among HF patients. Remote monitoring systems that track vital signs, symptoms, and medication adherence allow for the early detection of clinical deterioration, enabling timely interventions before decompensation occurs. Despite these benefits, telemedicine implementation faces several barriers, including technological limitations, reimbursement issues, digital literacy gaps, and challenges in integrating workflows. Future directions include developing standardized guidelines, designing patient-centered technologies, and establishing hybrid care models that combine virtual and in-person approaches. As healthcare systems worldwide seek more efficient and effective strategies for managing the growing population of individuals with HF, telemedicine offers a solution that may significantly improve patient outcomes and quality of life.

## Introduction

### Background

Heart failure (HF) is a significant global health burden characterized by the heart’s inability to pump sufficient blood to meet the body’s metabolic demands. It affects approximately 26 million people worldwide and is associated with substantial morbidity, mortality, and healthcare costs [1]. Despite advances in pharmacological and device therapies, HF continues to pose major challenges, with high readmission rates and a 5-year mortality approaching 50% in some populations [2]. Traditional in-person care models for HF management have inherent limitations, including inadequate monitoring between scheduled visits, delayed recognition of early decompensation signs, geographic barriers to specialist access, difficulties in optimizing medication regimens, and challenges in promoting patient self-management [3]. Telemedicine, broadly defined as the delivery of healthcare services using information and communication technologies (ICT) over a distance, has emerged as a promising approach to address these gaps. The World Health Organization first formalized the concept of telemedicine in 2007, emphasizing distance as a critical factor in its application; however, contemporary definitions now encompass any healthcare delivery that uses ICT for remote patient care, regardless of geographic proximity [4]. The coronavirus disease 2019 (COVID-19) pandemic significantly accelerated telemedicine adoption across all medical specialties, including cardiology, by necessitating alternative care delivery models [5]. For example, one large health system reported a 683% increase in virtual urgent-care visits over just 6 weeks in 2020 [6]. This rapid implementation demonstrated both the feasibility and potential benefits of virtual care approaches for managing cardiovascular disease, particularly chronic conditions such as HF.

### Objectives

This review examines the role and prospects of telemedicine in HF care, focusing on its historical development and definition, current clinical applications and supporting evidence, implementation challenges, and future directions.

## Ethics statement

As this study is a literature review, it did not require institutional review board approval or individual consent.

## Historical development of telemedicine

The evolution of telemedicine has paralleled advances in communication technology. Early forms of remote healthcare communication began in the 1840s with the invention of the telegraph, which enabled rudimentary long-distance medical consultations. The subsequent invention of the telephone expanded these capabilities; reports dating back to the early 1900s describe telephone-based diagnoses of croup and remote auscultation techniques [7]. The systematic development of telemedicine as a formal healthcare delivery approach gained momentum in the mid-20th century with several key milestones. In the 1960s, the National Aeronautics and Space Administration significantly advanced telemedicine by developing physiological monitoring systems for astronauts. These systems enabled the transmission of vital signs, including electrocardiograms (ECGs), from space to medical teams on Earth [8]. The emergence of the World Wide Web in the 1990s transformed telemedicine by broadening access beyond specialized fields such as aerospace medicine [9]. In 2003, the US Veterans Affairs healthcare system pioneered large-scale telemedicine implementation with its Home Telehealth program, targeting rural veterans with limited access to medical facilities [10]. By 2010, the Veterans Affairs had established a national telehealth center, initially focusing on mental healthcare for veterans affected by conflicts like the Iraq War and later expanding to comprehensive care models that included cardiovascular disease management [11]. The telemedicine market has experienced substantial growth, valued at approximately $21.2 billion in the United States alone and $49.8 billion globally in 2018, with projections suggesting a fivefold increase in the global market by 2026. Notably, the market encompasses both telemedicine products (hardware and software) and services (consultations and monitoring), with roughly equal distribution between these segments [12].

## Taxonomy and components of telemedicine

### Types of telemedicine interactions

The terms “telemedicine” and “telehealth” are often used interchangeably, but subtle distinctions exist. Telemedicine typically refers to remote clinical services, such as video consultations, telephone consultations, chat-based consultations, and remote diagnosis or data analysis. In contrast, telehealth covers a broader range of remote healthcare services, including not only telemedicine but also remote patient monitoring, remote surgery, remote diagnostics, electronic intensive care units, and remote clinical trials [13]. Telemedicine can be categorized based on synchronicity and the entities involved. Synchronous telemedicine refers to real-time interactions, where communication occurs instantly without delays. In a physician-to-patient setting, this includes live video or phone consultations during which a doctor provides immediate diagnosis and treatment recommendations. In a physician-to-physician context, it involves real-time discussions between medical providers who consult a specialist via video or phone call regarding a patient’s case [14].

Asynchronous telemedicine follows a store-and-forward approach, where medical data is recorded and reviewed later rather than in real time. In a physician-to-patient setting, this includes cases in which a patient uploads medical images or symptoms for later analysis by a doctor, who then provides medical advice. In a physician-to-physician context, a general practitioner may send a patient’s ECG or chest X-ray to a specialist, such as a radiologist or cardiologist, who reviews the data and offers expert opinion when available [15].

Hybrid telemedicine combines both synchronous and asynchronous approaches, leveraging real-time monitoring alongside delayed data analysis to provide more efficient and comprehensive care. In a physician-to-patient setting, a patient continuously records vital signs using wearable devices that automatically transmit and store data for later review. Physicians can then analyze trends over time and provide feedback asynchronously. In a physician-to-physician context, a doctor might consult an artificial intelligence (AI)–driven system to analyze stored medical data before discussing findings with a specialist in real time [16].

In HF management, various modalities have been applied that often combine remote monitoring of patient data with either synchronous or asynchronous provider feedback. This hybrid model shows promise in HF care by enabling continuous data collection with prompt intervention when necessary. For example, an HF patient can use a wearable device to continuously track vital signs such as heart rate, blood pressure, and oxygen levels. The data are recorded and transmitted, allowing physicians to analyze trends over time and detect early signs of HF. If a concerning pattern emerges, such as a gradual weight increase due to fluid retention, the physician can intervene before symptoms worsen, either through a video consultation or messaging. This approach is especially valuable in HF management, where rapid and proactive responses are critical to preventing acute decompensation and hospitalizations [17].

### Technical components of telemedicine systems

Effective telemedicine implementation for HF management requires a well-integrated technological infrastructure. Essential components include digital platforms, communication devices, and remote monitoring technologies. Digital platforms form the foundation of telemedicine services, ranging from basic data management systems to advanced platforms that incorporate AI for predictive analytics. These platforms facilitate secure data collection and storage, seamless integration with clinical workflows, communication between patients and providers, and the provision of analytics to support clinical decision-making [18]. Communication devices are vital for patient engagement in telemedicine, as they provide the primary means for remote interaction [19]. For example, the smartphone—one of the most well-known devices—is versatile, widely accessible, and serves as a key interface for telemedicine applications [20]. Remote monitoring devices enable health monitoring outside traditional clinical settings. These devices include connected blood pressure monitors, wireless weight scales, pulse oximeters, activity trackers, multi-parameter monitoring systems, and implantable hemodynamic sensors such as CardioMEMS [21].

For effective HF management, remote monitoring typically focuses on 3 key domains: risk factor monitoring, which tracks changes in blood pressure, glucose levels, and weight; medication adherence monitoring, which uses digital reminders and smart pillboxes to ensure patients follow their prescribed regimens; and symptom monitoring, which assesses clinical indicators such as dyspnea, fatigue, and edema [22,23]. The integration of these technological components creates a comprehensive telemedicine ecosystem that addresses the complex needs of HF patients throughout their care journey. By leveraging digital platforms, communication devices, and remote monitoring tools, telemedicine can enhance HF management, improve patient outcomes, and expand access to care (Tables 1, 2).

## Clinical evidence for telemedicine in HF management

### Reduced hospital readmissions and mortality rates

Remote monitoring systems have been shown to reduce hospital readmissions for HF patients. The TIM-HF2 trial, published in *The Lancet* in 2018, is one of the largest randomized studies of telemedicine in HF to date, involving approximately 1,500 participants [24]. This study implemented a structured telemedicine program that monitored body weight, blood pressure, heart rate, ECG, oxygen saturation, and self-reported health status. The results demonstrated a significant reduction in both all-cause mortality and HF-related hospitalizations in the intervention group compared to standard care (hazard ratio, 0.70; 95% confidence interval [CI], 0.50–0.96; P=0.0281). Subgroup analyses from this trial suggested particularly pronounced benefits among patients with diabetes mellitus as a comorbidity, underscoring the potential value of telemedicine for HF patients with multiple chronic conditions.

A comprehensive systematic review and meta-analysis examined the effectiveness of telemedicine, including home telemonitoring systems (hTMS), in HF management. This review analyzed 27 studies selected from an initial pool of 4,947 articles and demonstrated significant reductions in all-cause mortality (pooled odds ratio [OR], 0.65), cardiovascular mortality (OR, 0.68), and HF-related hospitalizations (OR, 0.77), particularly among patients with heart failure with reduced ejection fraction (HFrEF) [25]. Similarly, research on hTMS, which analyzed 65 non-invasive and 27 invasive studies involving 36,549 HF patients, revealed a 16% reduction in all-cause mortality, a 19% reduction in first HF hospitalization, and a 15% reduction in total HF hospitalizations [26]. These findings underscore that telemedicine plays a crucial role in improving outcomes by enhancing disease management, reducing hospital admissions, and lowering mortality rates in HF patients.

**Fig. 1.**
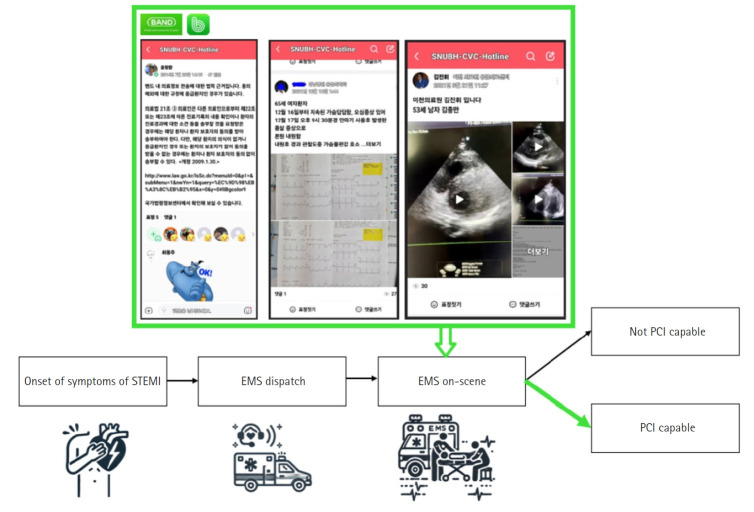
Telehealth in ST-elevation myocardial infarction using a social networking service (SNS) band to reduce the time for transfer. Adapted from Park et al. [27] under the CC-BY-NC license. STEMI, ST-segment elevation myocardial infarction; EMS, emergency medical service; PCI, percutaneous coronary intervention.

Utilizing advancements in communication technology, social networking service (SNS)-based emergency coordination has demonstrated potential for improving care efficiency. By enabling real-time communication among emergency cardiac care teams, this approach facilitates targeted resource allocation and optimized medication management. One study reported that implementing an SNS (BAND) for emergency cardiac teams significantly improved door-to-intervention times for patients with ST-elevation myocardial infarction. This was achieved by allowing emergency medical services to rapidly assess hospital availability, determine percutaneous coronary intervention capability, and coordinate patient transport in real time. The BAND enabled rapid pre-hospital communication, early diagnostic sharing, and streamlined preparation before patient arrival, ultimately reducing treatment delays and improving outcomes [27] (Fig. 1).

**Fig. 2.**
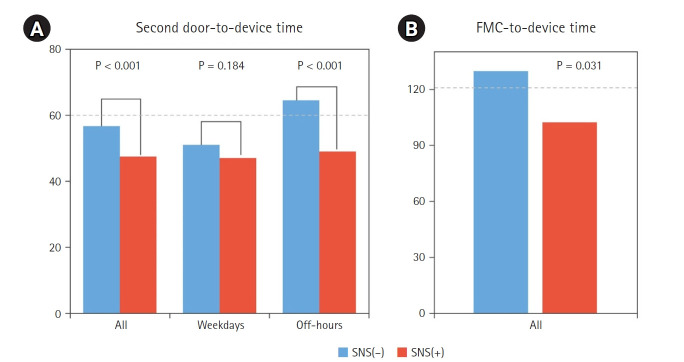
Impact of social networking service (SNS) on time to revascularization. Reproduced from Park et al. [27] under the CC-BY-NC license. FMC, first medical contact.

The door-to-device time was significantly shorter in the SNS (+) group compared to the SNS (–) group across all cases (P<0.001) and during off-hours (P<0.001), while no significant difference was observed on weekdays (P=0.184) (Fig. 2A). Furthermore, the first medical contact-to-device time was significantly shorter in the SNS (+) group (P=0.031), indicating that SNS utilization contributes to a more rapid treatment process (Fig. 2B).

In addition to non-invasive telemonitoring, implantable devices such as the CardioMEMS HF system enable direct measurement of pulmonary artery pressure. This approach facilitates earlier detection of worsening HF compared to conventional monitoring methods. By identifying clinical deterioration before the onset of severe symptoms, these devices support timely intervention, optimization of medical therapy, and more informed clinical decision-making. Collectively, these early responses can reduce hospital admissions and lead to significant cost savings in HF management through telemedicine [28,29].

### Improved medication adherence

Telemedicine significantly enhances medication adherence through integrated features designed specifically for HF patients. One key benefit is the optimization of guideline-directed medical therapy (GDMT), which comprises evidence-based pharmacological treatments—such as angiotensin receptor blockers, beta-blockers, mineralocorticoid receptor antagonists, and sodium-glucose cotransporter-2 inhibitors—recommended by clinical practice guidelines for HF management. Despite their proven benefits, GDMT is frequently underprescribed or administered at suboptimal doses due to barriers such as physician inertia, concerns about side effects, and inadequate follow-up [30,31]. Research indicates that telemedicine can overcome these challenges. In a randomized controlled trial involving 66 HF patients, those in the remote monitoring intervention group showed significant improvements in GDMT adherence compared to the standard care group. At the 6-month follow-up, the intervention group achieved a higher 4-GDMT score (64.6%) compared to 56.5% in the standard care group, demonstrating a significant enhancement in GDMT implementation. Although improvements in left ventricular ejection fraction and B-type natriuretic peptide levels did not reach statistical significance—likely due to the limited sample size—these findings support the potential of remote monitoring to improve GDMT quality and clinical outcomes in patients with HFrEF [32].

### Improved patient engagement

Telemedicine promotes patient engagement in HF management through multiple mechanisms. Virtual consultations offer opportunities for patient education and self-management support, allowing physicians to guide patients on symptom recognition, lifestyle modifications, and self-care strategies [33]. Structured symptom tracking using patient-reported outcome measures also helps patients become more aware of their condition and actively participate in monitoring their health [34]. Virtual cardiac rehabilitation programs further enhance engagement by providing home-based exercise sessions with remote monitoring, virtual classes on nutrition and lifestyle modification, online support groups, and telehealth coaching sessions [35,36].

**Fig. 3.**
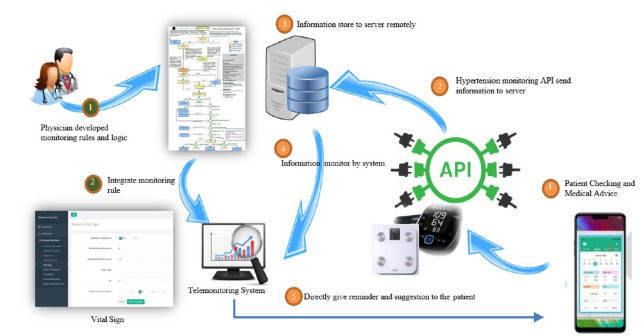
An advanced telemedicine system incorporating artificial intelligence (AI) for heart failure patients’ home care. Reproduced from Yoon et al. [37] under the CC-BY license. API, Application Programming Interface.

A multi-center study conducted across 7 South Korean hospitals developed and tested an advanced telemedicine system incorporating AI-enhanced predictive algorithms [37]. This comprehensive platform included a patient smartphone application, connected weight and blood pressure monitoring devices, and a provider dashboard with automated alerts. The smartphone application allowed patients to track symptoms—including dyspnea, fatigue, edema, and palpitations—using a structured scale, while also logging vital signs such as blood pressure, heart rate, weight, and body water. The system integrated Bluetooth-connected monitoring devices to ensure automated, real-time data collection (Fig. 3).

**Fig. 4.**
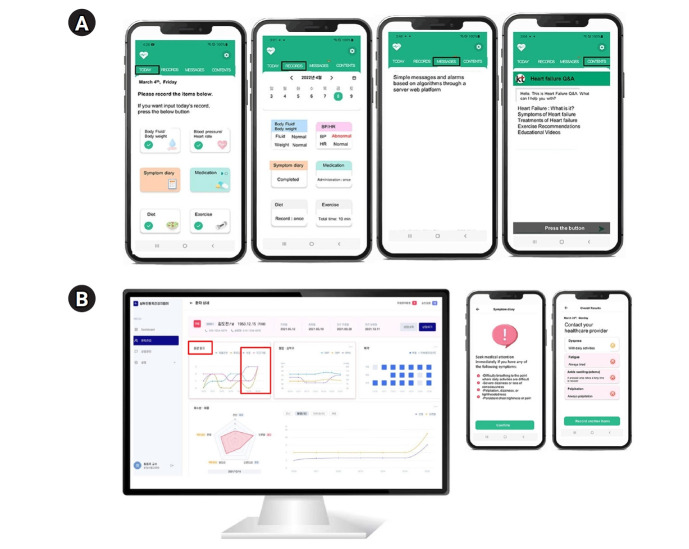
Telehealth in heart failure using an artificial intelligence (AI) platform. (A) How the AI platform appears on the smartphone screen and (B) the provider dashboard and patient alert system. Reproduced from Yoon et al. [37] under the CC-BY license.

Additionally, AI-assisted dietary analysis enabled sodium intake estimation via image recognition to support better dietary management. Beyond self-monitoring, the app provided personalized feedback on medication adherence and symptom trends (Fig. 4A). A clinical decision support system continuously analyzed patient data to detect significant health changes, generating alerts that prompted patients to assess their condition and seek medical attention when necessary. Furthermore, educational resources on HF management were available to improve patient knowledge and self-care practices (Fig. 4B).

In a randomized evaluation involving approximately 130 patients followed for 4 weeks, the intervention group demonstrated significant improvements in dyspnea symptom scores compared to the control group. This evidence shows that technology-enabled care can yield measurable clinical improvements while simultaneously increasing patient engagement and empowering individuals to take a more active role in managing their condition.

## Challenges and barriers to telemedicine implementation

### Technological barriers and infrastructure

A fundamental challenge is the availability and reliability of technology. Telemedicine depends on stable internet connections and sufficient bandwidth, which may be lacking in rural areas or low-resource settings. Patients in regions with poor connectivity or those who cannot afford broadband may be unable to effectively participate in video visits or continuous data transmission. Even when connectivity is available, ensuring interoperability between various devices and platforms is difficult [38]. In HF telemonitoring programs, patients might use different brands of blood pressure cuffs, weight scales, and wearables; integrating these diverse data streams into a coherent platform for physicians is technically complex [39]. Lack of standardization can result in systems that do not communicate effectively, leading to fragmented information. Moreover, data security and privacy are major concerns. Transmitting personal health information over networks raises the risk of data breaches or unauthorized access, and both physicians and patients may worry about the confidentiality of sensitive medical information. While strict adherence to security protocols (such as encryption, secure servers, and Health Insurance Portability and Accountability Act-compliant software) is essential, not all telemedicine solutions meet these standards [40].

### Regulatory and reimbursement issues

Health policy and reimbursement frameworks have not always kept pace with telemedicine technology. Historically, many insurance systems provided limited or no reimbursement for telemedicine services, discouraging investment in virtual care. For example, before 2020, Medicare in the United States only reimbursed telehealth for patients in certain rural areas or specific circumstances, often at lower rates than in-person visits. Licensing requirements also posed challenges; a physician must typically be licensed in the state where the patient is located, complicating cross-state telemedicine even over short distances [41]. This fragmentation meant that a patient seeking consultation from a renowned HF specialist in another state via telemedicine could face legal barriers unless the physician obtained multiple state licenses. Malpractice coverage for telehealth was another uncertain area. Although many of these regulatory constraints were relaxed during the COVID-19 pandemic—leading to a significant uptick in telemedicine use—it remains uncertain whether these favorable policies will persist long-term. If reimbursement reverts to pre-pandemic models or cross-state licensing flexibility is withdrawn, providers may scale back telehealth offerings. Uncertainty in payment models is a barrier, and healthcare organizations may hesitate to invest in telemedicine programs if financial sustainability is unclear [42]. Additionally, telemedicine raises questions regarding interstate practice, liability laws across jurisdictions, and even issues like the remote prescribing of controlled substances [43]. Policymakers and regulatory bodies are actively addressing these issues, yet the absence of universally adopted telemedicine guidelines and inconsistent policies across regions continues to hinder widespread adoption. Continued advocacy is required to ensure that providers are adequately compensated for telemedicine services and that patients receive insurance coverage comparable to in-person care.

### Patient factors: digital literacy and trust

Telemedicine inherently requires patients to engage with digital technology, introducing challenges related to patient capability and access. Digital literacy—the ability to use devices and navigate digital interfaces—varies widely among patient populations [44]. Older patients, who comprise a large proportion of those with HF, may be less familiar with smartphones, computers, or even basic cell phone functions. Additionally, some patients may experience cognitive impairments or visual/hearing deficits that complicate the use of telehealth apps. Socioeconomic factors also play a role; patients with lower incomes or education levels may lack access to appropriate devices or struggle with the usability of health-related applications. Consequently, vulnerable populations risk being excluded from the benefits of telemedicine, potentially exacerbating existing health disparities—a phenomenon often referred to as the digital divide. Another barrier is trust and personal preference. Some patients are skeptical of remote care, feeling that virtual visits are not as thorough as in-person consultations [45]. Concerns may include discomfort discussing sensitive issues via video or fears that clinical details may be overlooked. Establishing trust in telemedicine requires assuring patients that their needs will be fully addressed and emphasizing that remote care complements, rather than replaces, face-to-face interactions when clinically appropriate.

### Physician factors: workflow challenges

**Fig. 5.**
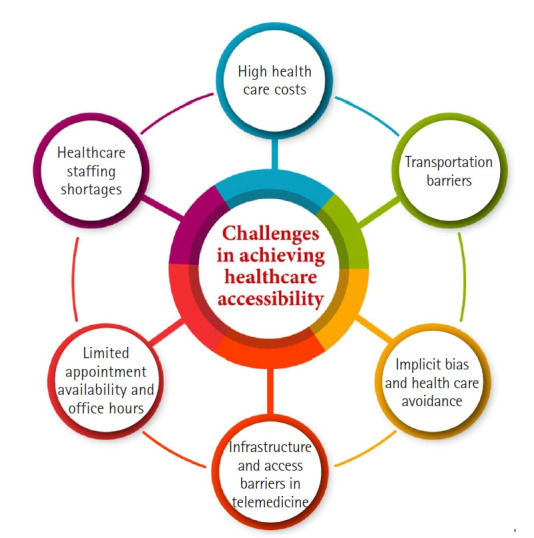
Various factors impeding access to healthcare, each contributing to the complex landscape of healthcare accessibility. Adapted from Anawade et al. [47] under the CC-BY license.

Integrating telemedicine into HF management presents significant workflow challenges for physicians. They must adapt to video visit platforms, manage electronic patient communications, and interpret continuous remote monitoring data—often leading to “alert fatigue” when numerous patients transmit daily readings. In addition, many physicians require training in virtual examination techniques and must overcome initial resistance to this modality, which some feel lacks the personal connection of traditional care. These challenges are often mitigated through the use of smart alert algorithms and, in some cases, by deploying dedicated telemonitoring personnel—resources that may not be readily available in all clinical settings [46]. Various factors impede access to healthcare, each contributing to the complex landscape of healthcare accessibility [47] (Fig. 5).

## Future perspectives and recommendations

### Standardization of telemedicine protocols and guidelines

A key recommendation is the development of standardized guidelines and policies for telemedicine in HF. Professional societies and public health authorities are already working toward this goal. The American Heart Association recently published a scientific statement outlining best practices for telehealth in cardiovascular and stroke care [48]. Such guidelines provide a structured framework for determining when and how to use telemedicine, along with clearly defined standards for quality, safety, privacy, and data security, as well as metrics for evaluating outcomes. Regulators are encouraged to harmonize policies across regions—for example, by simplifying licensure for telemedicine across state or national borders—to facilitate access to expert care regardless of patient location. Furthermore, integrating telemedicine documentation and data into existing health information systems is crucial for continuity of care. Initiatives such as creating standardized telehealth visit codes and telemonitoring data fields in electronic health records can help make telemedicine a seamless part of clinical workflows. In addition, reimbursement models should be formalized to ensure permanent coverage of telehealth services at parity with in-person care, particularly given their demonstrated clinical efficacy.

### Patient-centered technology development

A critical future focus for telemedicine is to improve accessibility and acceptability across diverse patient populations. This effort requires involving patients in telehealth system design, developing user-friendly interfaces, and providing educational resources such as tutorial videos, helplines, and peer mentoring to improve digital literacy. To address equity concerns, healthcare systems should consider device lending programs and internet access support for underserved communities, while also implementing culturally sensitive adaptations in language and health education. Rather than adopting a one-size-fits-all approach, providers should assess individual circumstances and preferences—offering high-tech monitoring for tech-savvy patients while maintaining low-tech options like phone calls for others. Continuous collection of patient experience data will help refine these services, ensuring that telemedicine’s benefits extend to all populations, including those in remote or historically underserved communities, and ultimately preventing digital disparities in HF care outcomes [49].

### Hybrid care models

**Fig. 6.**
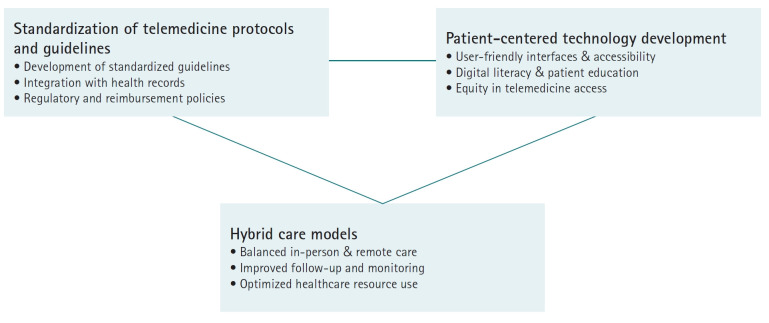
Future perspectives and recommendations for telemedicine in heart failure (Drawn by the author).

Experts recommend adopting hybrid models that blend in-person and remote care rather than treating telemedicine as a complete replacement for traditional approaches. For HF patients, this means scheduling in-person visits for critical moments, such as initial diagnosis or when detailed physical examinations are necessary, while using telemedicine for routine monitoring and follow-ups. This balanced approach can increase healthcare capacity by allowing physicians to manage larger patient panels while providing more frequent touchpoints without overwhelming clinic schedules. Telemedicine can also enhance after-hours support, potentially preventing emergency department visits through remote assessment and medication adjustments, thereby reducing acute care burdens on healthcare providers (Fig. 6).

## Conclusion

HF requires continuous management and is associated with high mortality, making telehealth an essential tool for effective care. This review has examined substantial evidence supporting telemedicine’s effectiveness in improving clinical outcomes, optimizing medication therapy, and providing personalized care. Despite its effectiveness and growing implementation, significant challenges remain and must be addressed to realize telemedicine’s full potential. When thoughtfully implemented with attention to evidence and integrated with existing care systems, telemedicine has the potential to significantly improve the quality of HF management.

## Figures and Tables

**Table 1.** Telemedicine components

Components	Telehealth ⊃ Telemedicine
Digital platform	- Secure data collection and storage
	- Clinical workflow integration
	- Provider-patient communication support
	- Analytics for decision-making
	- AI for predictive capabilities
Communication device	- Smartphones for application interface
	- Primary remote interaction tools
	- Patient engagement enablers
	- Secure messaging capabilities
	- Virtual visit technology
Remote monitoring technology	- Risk factor monitoring
	- Medicine adherence
	- Symptom monitoring

AI, artificial intelligence.

**Table 2.** Telemedicine types

Types	Synchronous+	Asynchronous=	Hybrid
Visits	Virtual visits: Direct physician-patient interaction with immediate assessment and treatment recommendations	eVisits: Patient-submitted health data and symptoms reviewed by physicians with delayed response and treatment plans	Remote monitoring: Continuous tracking of vital signs and symptoms synchronously through wearable devices with asynchronous physician review
Consult	Virtual consults: Live video consultations between physicians for immediate specialist input on patient cases	eConsults & second opinions: Medical data and images forwarded to specialists for expert review and recommendations when available	Predictive analytics: AI-driven analysis of patient data to identify deterioration patterns and risk factors, enabling proactive interventions

AI, artificial intelligence.
